# Effects of photodynamic therapy on *Enterococcus faecalis* biofilms

**DOI:** 10.1007/s10103-015-1749-y

**Published:** 2015-04-28

**Authors:** L. López-Jiménez, E. Fusté, B. Martínez-Garriga, J. Arnabat-Domínguez, T. Vinuesa, M. Viñas

**Affiliations:** Laboratory of Molecular Microbiology and Antimicrobials, Department of Pathology and Experimental Therapeutics, Medical School, IDIBELL-University of Barcelona, Barcelona, Spain; Department of Dentistry, Dentistry School, IDIBELL-University of Barcelona, Barcelona, Spain

**Keywords:** Atomic force microscopy, Confocal laser scanning microscopy, Photodynamic therapy, *Enterococcus faecalis*, Surface roughness

## Abstract

Microbial biofilms are involved in almost all infectious pathologies of the oral cavity. This has led to the search for novel therapies specifically aimed at biofilm elimination. In this study, we used atomic force microscopy (AFM) to visualize injuries and to determine surface roughness, as well as confocal laser scanning microscopy (CLSM) to enumerate live and dead bacterial cells, to determine the effects of photodynamic therapy (PDT) on *Enterococcus faecalis* biofilms. The AFM images showed that PDT consisting of methylene blue and a 670-nm diode laser (output power 280 mW during 30 s) or toluidine blue and a 628-nm LED light (output power 1000 mW during 30 s) induced severe damage, including cell lysis, to *E. faecalis* biofilms, with the former also causing an important increase in surface roughness. These observations were confirmed by the increase in dead cells determined using CLSM. Our results highlight the potential of PDT as a promising method to achieve successful oral disinfection.

## Introduction

A microbial biofilm is a three-dimensional, complex structure attached to a surface or interface and comprising microorganisms embedded in an extracellular polymeric matrix [1]. Although laboratory studies of biofilm formation and structure commonly make use of monospecies biofilms, in nature, biofilms are frequently formed by more than one species and in some cases by hundreds of species. An excellent example is oral biofilms, referred to as dental plaques [3, 4]. These typically contain an enormous variety of bacterial species, many of which are responsible for infections in the oral cavity and even elsewhere in the body [2]. Dental caries, periodontal diseases, endodontic infections, and numerous pathologies beyond the oral cavity have been attributed to the proliferation of oral bacteria and their ability to form and participate in stable polymicrobial biofilms.

Microbes living in a biofilm are subjected to environmental conditions that promote behaviors clearly different from those of planktonic forms. Transcriptomic studies have identified several genes that are overexpressed in sessile vs. planktonic bacteria, whereas other genes are downregulated [5]. In addition, bacteria in biofilms are typically much more resistant to antimicrobials than their planktonic counterparts [6], although the latter are used in the clinical testing of susceptibility.

Oral bacteria grow exclusively (or almost) in biofilms. Thus, the main goal of oral disinfection is biofilm destruction, together with elimination of the remaining viable bacteria. Because of its anatomical complexity, the root canal system acts as a reservoir of several bacterial species that grow in biofilms. Mechanical disruption and antimicrobial therapies are currently the most frequently used methods to treat and eliminate oral biofilms; however, the effectiveness of these strategies is limited by the emergence of resistant microorganisms and the common persistence of a small proportion of viable bacteria, both of which can largely if not completely regenerate the community [7]. Consequently, there has been extensive research into the development of alternative therapeutic methods, such as laser light treatments and photodynamic therapy (PDT) [8, 9]. In the former, high-power lasers are used together with an intracanal optical fiber first to kill the microorganisms by means of a photothermal effect and then to disinfect areas unreachable by traditional endodontic treatments [10–12]. However, high-power lasers may cause thermal injuries to dental tissues, such as dentin carbonization and cratering, root resorption, cementum melting, and periradicular necrosis [13–15]. Thus, PDT, in which low-power lasers drive photochemical reactions, has been suggested as a promising approach to fight oral infections without the undesirable effects associated with temperature increases. PDT is based on the use of photosensitizers, molecules that are activated by light in the presence of air. Activation leads to the generation of highly reactive singlet oxygen and free radicals that have cytotoxic effects on living cells [2]. Singlet oxygen is a diamagnetic form of oxygen and is responsible for the damaging effects of sunlight on organic materials. It is usually produced by means of photosensitizer pigments (most of them vital stains) and is stable for over an hour at room temperature. PDT has been used to kill cancer cells and bacteria, by exploiting their sensitivity to singlet oxygen. In principle, photosensitizers can penetrate both gram-positive and gram-negative bacteria, without affecting host cell viability [16]. Moreover, bacteria are unlikely to develop resistance to repeated photosensitization [17]. Several studies have reported the successful use of PDT in reducing bacterial counts in the root canal system, recommending it as an adjunctive antimicrobial procedure in conventional endodontic treatment [18–22]. It has been pointed out that PDT is as effective as conventional 5 % NaOCl irrigation against *Enterococcus faecalis* [23]. Moreover, the efficacy of PDT on biofilms seems to be strain dependent [24].

In this study, we examined the effects of PDT on biofilms of *Enterococcus faecalis*, a gram-positive bacterium resistant to some antibiotics and frequently found in the oral cavity of patients who have undergone root canal treatment. Our combined approach consisted of atomic force microscopy (AFM), confocal laser scanning microscopy (CLSM), and surface roughness determinations. The results highlight the potential of PDT to achieve successful oral disinfection.

## Material and methods

### Bacterial strain, culture conditions, and biofilm formation

*E. faecalis*, American Type Culture Collection (ATCC) 29212, was grown overnight in 20 ml of trypticase soy broth (Scharlau, Barcelona, Spain) at 37 °C with orbital shaking at 250 rpm. The culture was used to inoculate 24-well culture plates containing 2 ml of growth medium to yield a bacterial concentration of 10^6^ colony-forming units (cfu)/ml. Each well contained a Thermanox coverslip previously coated with a 0.01 % (*w*/*v*) poly-l-lysine hydrobromide (Sigma-Aldrich, Dorset, UK) solution to enhance bacterial cell adhesion and to prevent biofilm removal during the experiments. The plates were incubated at 37 °C for 24 h under gentle shaking (60 rpm) to allow biofilm formation.

### Photosensitizers and light sources

The photosensitizers tested in this study consisted of two dyes, toluidine blue O (TBO), at 0.1 mg/ml, and 3,7-bis(dimethylamino)-phenazathionium chloride trihydrate (methylene blue, MB), at 0.005 % (*w*/*v*) in phosphate-buffered saline containing hydroxymethylcellulose as a mucoadhesive viscosity agent (Periowave, Ondine Biopharma, Vancouver, BC, Canada). Both TBO and MB are commonly used in oral antimicrobial PDT. Their activities as potent photosensitizers against gram-negative and gram-positive bacteria were previously documented [2].

A light-emitting diode (LED) lamp (FotoSan; CMS Dental, Copenhagen, Denmark), emitting in the red spectrum with a peak at 628 nm (620–640 nm), was used as the light source together with TBO. The LED lamp has an output power of 1000 mW for 30 s, total energy delivered was 30 J; surface at the end of the fiber was 6 mm diameter, and an energy density of 106.4 J cm^−2^. For MB, diode laser light (Periowave; Ondine Biopharma, Vancouver, Canada) emitting at a wavelength of 670 nm served as the light source. It has an output power of 280 mW during 30 s, total energy delivered was 8.4 J, and an energy density of 271 J cm^−2^.

### PDT

The biofilms were gently washed with distilled water to remove nonadherent bacteria. The experimental conditions were (i) biofilms sensitized with TBO in darkness for 1 min, (ii) biofilms sensitized with TBO in darkness for 1 min and then treated with LED for 30 s, (iii) biofilms sensitized with MB in darkness for 1 min, and (iv) biofilms sensitized with MB in darkness for 1 min and then exposed to diode laser for 30 s. The controls consisted of (i) biofilms treated neither with photosensitizers nor with light sources, (ii) biofilms treated only with the diode laser for 30 s, and (iii) biofilms treated only with the LED lamp for 30 s. After treatment, all of the biofilms were gently washed with distilled water and visualized by AFM and CLSM.

### AFM imaging

Samples were imaged in air using an atomic force microscope XE-70 (Park Systems, Korea). All images were collected in noncontact mode using pyramidal-shaped silicon cantilevers with a spring constant of ±40 N m^−1^, a resonance frequency of ±300 kHz, and their upper sides coated with aluminum to enhance the reflectivity of the laser beam. AFM images were simultaneously acquired with several scan sizes (100, 25, and 6.25 μm^2^) at a scan rate of 0.3–0.5 Hz. Data acquired during surface scanning were converted into images of topography, amplitude, and phase and then analyzed using XEP and XEI software (Park Systems, Korea). Topography images reveal the shape and structure of the sample as well as surface differences. Amplitude images highlight the sample outline and allow visualization of fine surface details. Phase images show variations in the elasticity and viscoelasticity of the sample.

### CLSM imaging

Biofilms on the Thermanox coverslips were washed three times with distilled water to remove loose bacteria and then stained using the LIVE/DEAD *BacLight* bacterial viability kit (Molecular Probes, Eugene, OR). In this system, live bacteria stain with Syto 9 to produce a green fluorescence whereas bacteria with compromised membranes stain with propidium iodide to produce a red fluorescence. Images of the double-stained biofilms were acquired using a Leica TCS-SL filter-free spectral confocal laser scanning microscope (Leica Microsystems, Mannheim, Germany) equipped with a 488-nm argon laser and 543- and 633-nm He/Ne lasers (Centres Científics i Tecnològics, Universitat de Barcelona, Barcelona, Spain) and a × 63 oil immersion objective (1.4 numerical aperture) zoom 1, where the *x*, *y*, and *z* voxel size corresponded to 0.23 × 0.23 × 0.4 μm with an image resolution of 1024 × 1024 pixels. The pinhole size was kept at the minimum setting (1.0–1.08 AU). Image saturation was prevented by lowering the gain and offset in the brightest signal. Sequential scanning was carried out for each channel. CLSM images were analyzed by using ImageJ software (National Institutes of health, Bethesda, MD, USA). A threshold selection method was created to distinguish between one and two bacteria. Alive and dead bacteria percentages were calculated from the total number of bacteria. Values (percentages) were arcsine transformed. Furthermore, data were analyzed by Kolmogorov-Smirnov test and Levene one-way ANOVA tests. *P* values lower than 0.05 were considered as statistically significant.

### Surface roughness

AFM was also used to measure the surface roughness of the treated and untreated biofilms. The roughness average (*R*_*a*_), defined as the average distance from the roughness profile to the center plane of the profile, was calculated from the acquired topography images for every scan size and treatment.

### Bacterial enumeration

The CLSM images were analyzed using ImageJ (National Institutes of Health, Bethesda, MD, USA) to enumerate viable and dead bacteria, differentially stained as described above. A thresholding procedure was established for every image, and a watershed separation was applied to separate clusters of bacteria. Percentages of live and dead bacteria in every treatment were determined.

## Results

### Visualization of PDT effectiveness

**Fig. 1:**
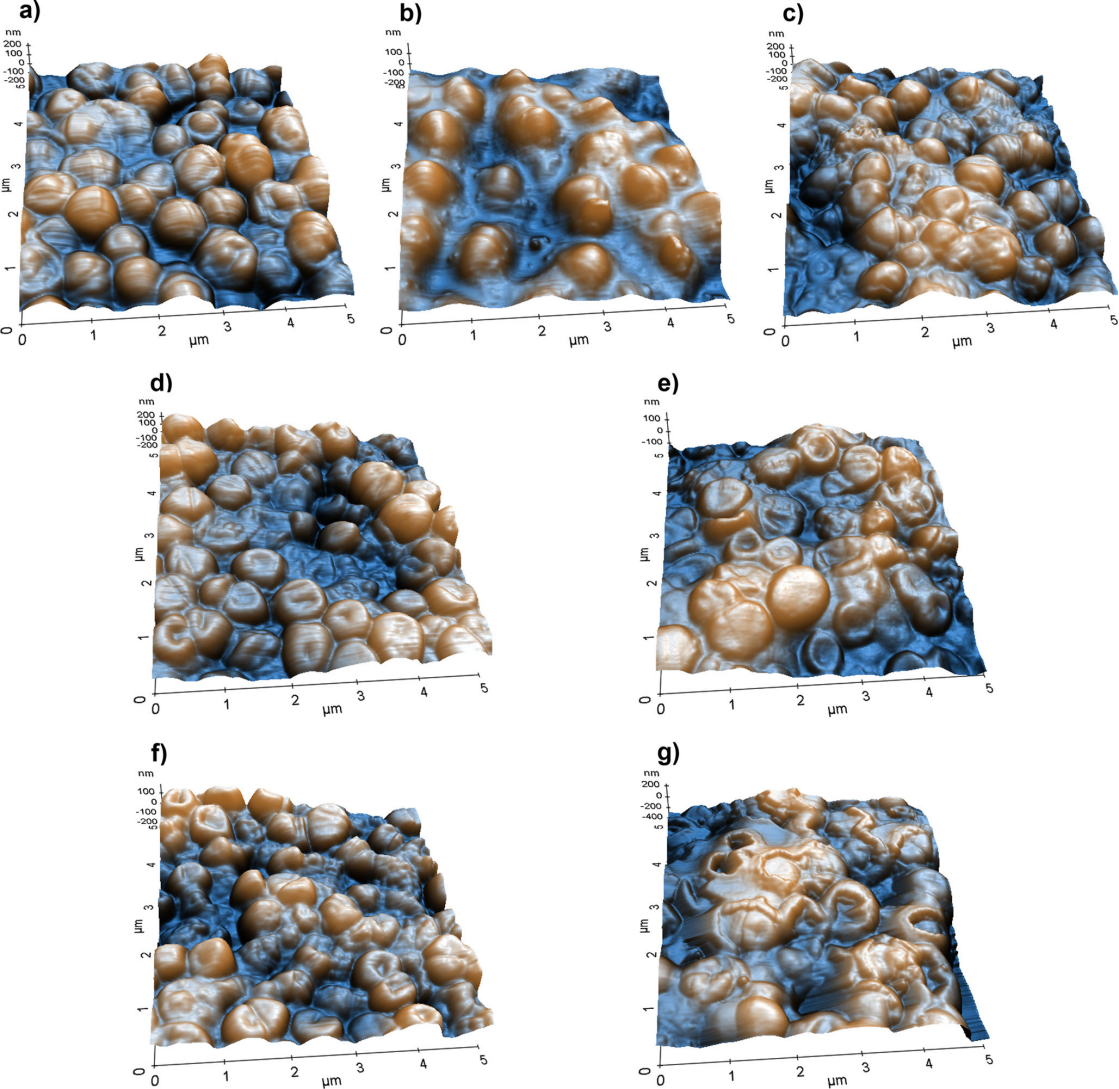
AFM 3D topography images of *E. faecalis* biofilms: untreated (**a**), exposed to LED for 30 s (**b**), exposed to diode laser for 30 s (**c**), sensitized 1 min with TBO (**d**), sensitized 1 min with MB (**e**), sensitized 1 min with TBO and exposed to LED for 30 s (**f**), and sensitized 1 min with MB and exposed to diode laser for 30 s (**g**). Scan size = 25 μm^2^

Representative AFM images of treated *E. faecalis* biofilms are shown in Fig. 1. An analysis of the AFM topography 3D-images showed that PDT induced severe morphological and surface alterations of the biofilms as well as a broad spectrum of injuries to the resident bacterial cells, whereas in the untreated biofilms, *E. faecalis* retained its typical coccoid shape (Fig. 1a). In the biofilms treated with TBO (1 min) plus LED (30 s), bacterial wall destruction, loss of the typical cell morphology, and leakage of the intracellular contents were observed (Fig. 1f). The injuries to biofilms treated with MB (1 min) plus diode laser (30 s) were similar (Fig. 1g) but much more apparent. Neither TBO nor MB alone was able to induce noticeable morphological alterations (Fig. 1d, e), as only a small proportion of bacterial cells were even slightly affected. Conversely, light therapy in the absence of the dyes caused slight changes in biofilm topography (Fig. 1b, c).

### Surface roughness

**Fig. 2:**
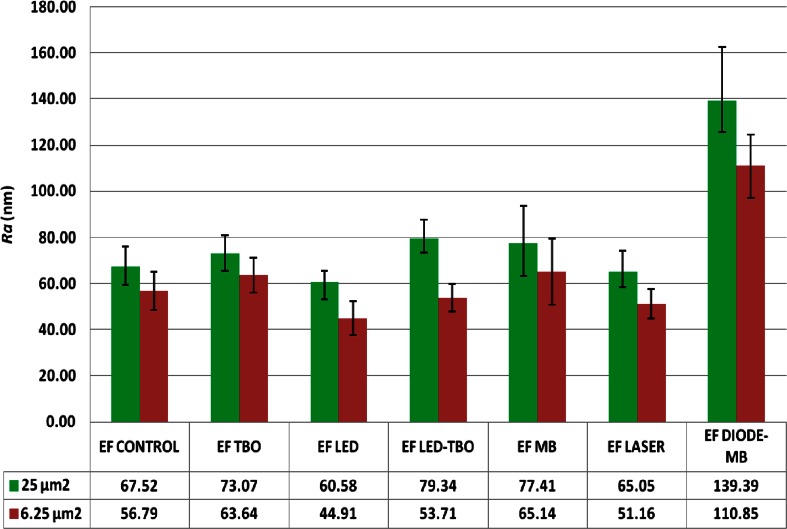
Graphical representation of surface roughness (*R*_a_) in nanometers, according to the different treatments tested and surface scan sizes. *Bars* represent the standard error of the mean. The mean surface roughness values are shown in the table below

Changes in the surface roughness of treated vs. untreated biofilms can be numerically expressed using the XEI software and processing the topography images previously obtained at scan sizes of 25 and 6.25 μm^2^. Figure 2 shows the mean surface roughness values (*R*_*a*_) of control and treated *E. faecalis* biofilms. PDT-treated biofilms had high surface roughness values. By contrast, the roughness values measured after treatment of the biofilms with the dyes or with either light source alone were not significantly modified.

### CLSM

**Fig. 3:**
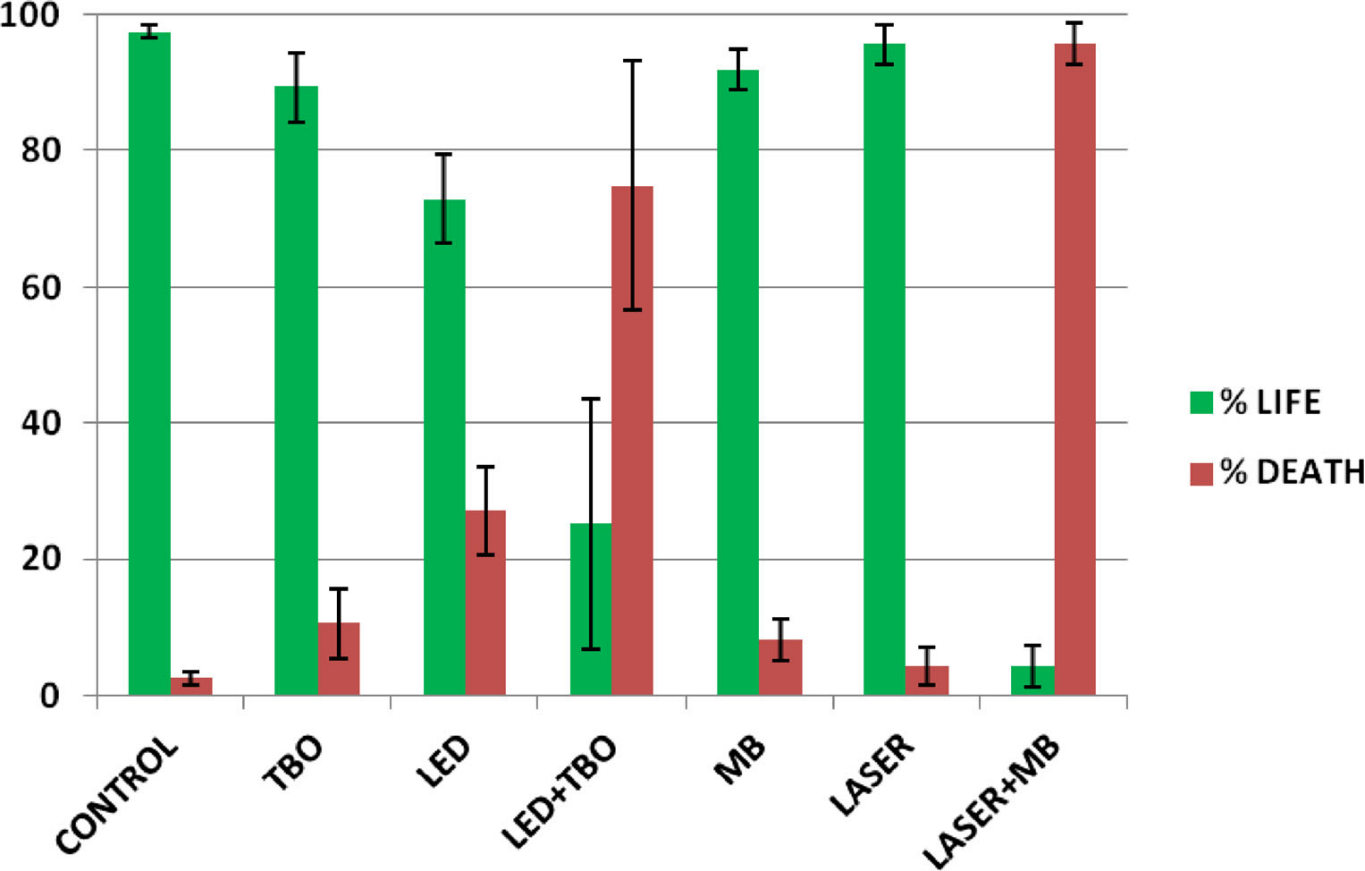
Graphical representation of living and dead bacteria, according to the different treatments tested. *Bars* represent the standard error of the mean

Enumeration of the viable and dead bacteria for every treatment showed a significant increase in bacterial death in *E. faecalis* biofilms treated with PDT. Neither of the photosensitizers alone resulted in significant bacterial killing, as the proportion of living bacteria in either case was almost identical to that in the negative controls (about 2 %). LED treatment in the absence of photosensitizer had a slight lethal effect, with approximately 24.2 % of the individual cells exhibiting red fluorescence. Diode laser treatment alone, at least at the power tested, was unable to kill bacteria. On the contrary, PDT caused significant bacterial injury, with more than 95 % lethality in the case of diode laser plus MB and 79 % in the case of LED plus TBO (Fig. 3).

**Fig. 4:**
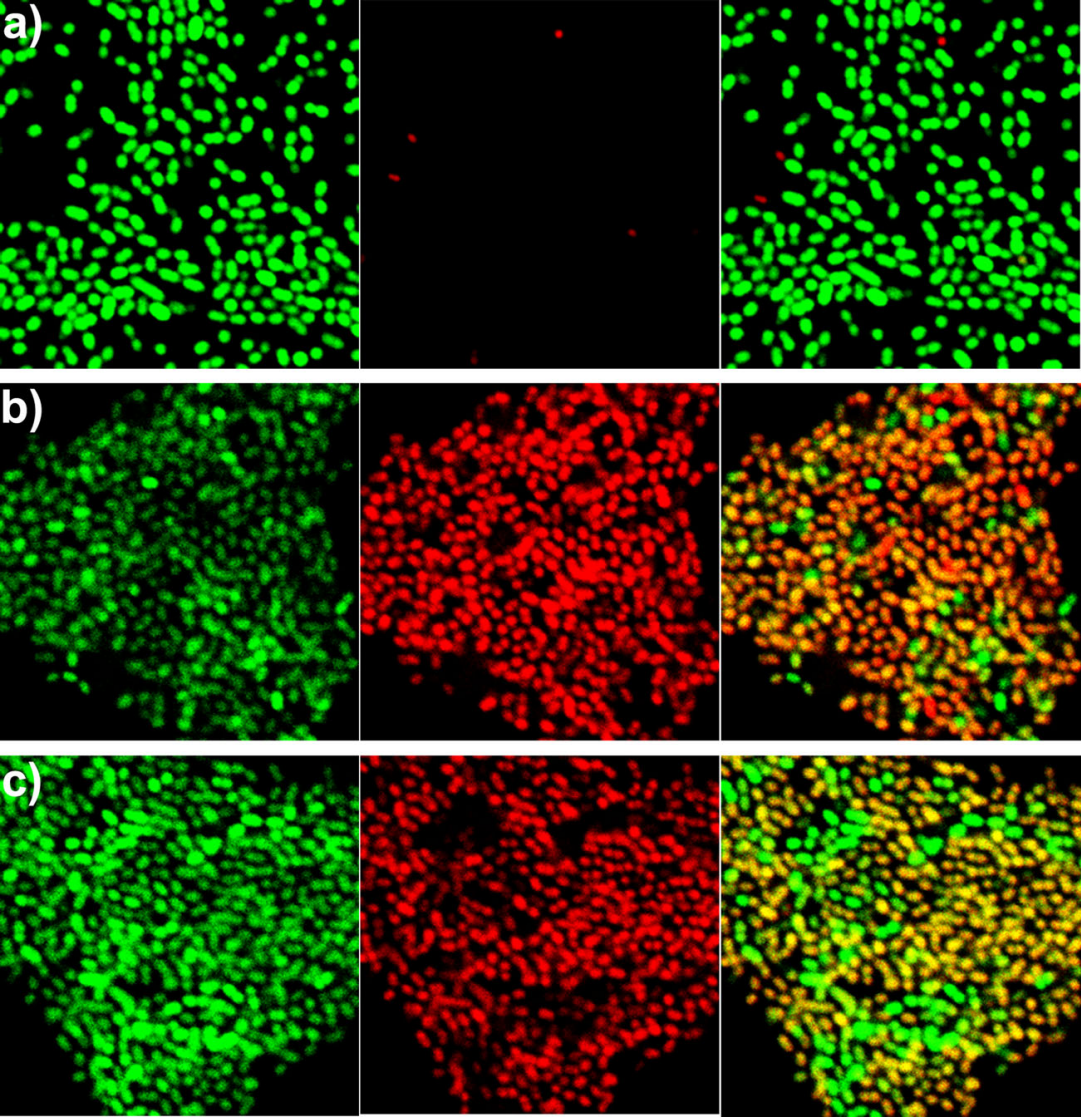
CLSM images of *E. faecalis* biofilms: untreated (**a**), treated with LED plus TBO (**b**), and treated with diode laser plus MB (**c**). Viable (*green*) bacteria (*left*), dead (*red*) bacteria (*middle*), and viable and dead bacteria (*right*). *Scale bar* = 10 μm

The results of CLSM showed predominance of red fluorescence indicating damaged biofilm cells following PDT treatments in comparison with the control showing green color dominance in biofilm. Representative CLSM images of untreated and PDT-treated biofilms are shown in Fig. 4. On the double-fluorescence images of living plus dead bacteria, the majority of the cells in the untreated biofilms stained green (Fig. 4a), indicating a high level of bacterial viability. In biofilms treated with LED plus TBO (Fig. 4b) or diode laser plus MB (Fig. 4c), most of the bacteria stained red, indicating significant bacterial killing. In these PDT-treated images, orange-staining bacteria were considered to be damaged cells. Table 1 shows statistical analysis of the results of CLSM experiments.

**Table 1:** Multiple comparisons of percentages of alive/dead bacteria after treatments

	Control	TBO	MB	LED	LASER	LED + TBO
TBO	0.802					
MB	0.916	0.999				
LED	0.068	0.534	0.385			
LASER	0.999	0.864	0.953	0.086		
LED + TBO	*0.000*	*0.002*	*0.002*	*0.0048*	*0.000*	
LASER + MB	*0.000*	*0.000*	*0.000*	*0.010*	*0.000*	0.973

Statistically significant values are in italics

## Discussion

In the biofilm mode of growth, microorganisms firmly attached to a surface or interface are enclosed in an extracellular polymeric matrix formed by polysaccharides, nucleic acids, proteins, water, and cell debris. This matrix offers protection against host defenses and often restricts the penetration of antimicrobial agents [1, 25]. Thus, for infections of the oral cavity, in which the causative agents typically reside in biofilms, conventional treatments are often ineffective. A therapeutic approach is therefore needed that focuses on biofilm removal, the eradication of persistent cells, and the decontamination of oral surfaces. PDT has been used to eliminate bacterial as well as cancer cells. Recently, it has emerged as an alternative in the removal of oral biofilms and thus in the prevention or amelioration of infections of the oral cavity.

In the present work, we used three different approaches, AFM, CLSM, and surface roughness determination, to study the efficacy of PDT in the elimination of *E. faecalis* biofilms formed on Thermanox coverslips. *E. faecalis* is commonly found in the root canal system of the teeth, where its biofilm-type growth has been documented.

AFM is a powerful tool that, with easy sample preparation, provides high-resolution imaging of microbiological systems [26], individual microbial cells [27], and microbial biofilms [28]. It has also been used to study the mechanical and adhesive properties of microbial cells [26], to assess surface properties such as roughness, both in air and in liquid [29], to evaluate morphological effects of treatments, including those targeting microorganisms [30], and to study microbial cell processes and interactions [31]. However, a limitation of AFM is that under physiological conditions, some structures and morphological features, such as flagella or biofilm matrix components, are poorly visualized by liquid imaging [27, 32–34] because of deficient adhesion of the microbial cells to the substrate. This, in turn, generates sufficient noise during scanning such that image quality is compromised [35, 36]. In our study, we used air-dried samples since AFM imaging of dried microbial preparations is well established [33, 37]. The advantages include easy sample preparation and high-resolution imaging of microbial cell surfaces [38, 39]. AFM visualizations in air are commonly used to evaluate morphological changes in treated vs. nontreated microbial surfaces [34, 39–44]. Thus, it was the method of choice in the analysis of our air-dried treated and untreated biofilms. Imaging of the latter revealed their normal morphology and low nano-roughness values throughout the incubation period. This result confirmed that the significant morphological alterations and surface injuries observed in the treated samples were produced by PDT and were not sample processing artifacts. Specifically, AFM of the sensitized biofilms revealed that with TBO or MB alone only, a small proportion of bacterial cells showed bacterial wall perturbations (Fig. 1d, e). Similar alterations were produced after treatment by light in the absence of dyes (Fig. 1b, c). However, *E. faecalis* biofilms exposed to PDT (Fig. 1f, g), and particularly those treated with MB and diode laser, showed severe alterations.

A relevant parameter to characterize surface morphology is the determination of surface roughness, expressed as the arithmetic average roughness *R*_*a*_ [45]. An increase in cell surface roughness is indicative of a distorted cell morphology, bacterial wall destruction, and leakage of cellular contents, all of which were observed in the PDT-treated biofilms, thus confirming the AFM findings. The surface roughness data also showed that the greatest damage occurred in biofilms exposed to MB and diode laser (Fig. 2). In irradiated cells, photosensitizers may cause alterations to membrane integrity and thus also damage cytoplasmic components [46]. These results support the use of PDT to destroy *E. faecalis* biofilms.

CLSM was used to assess bacterial viability in biofilms subjected to PDT or to the photosensitizers or light alone and it confirmed our AFM results. With the double-staining method, we were able to distinguish between bacteria with intact (green) and damaged (red/orange) membranes [24]. CLSM images of the untreated biofilm showed that most of the bacteria stained green, indicating their viability (Fig. 4a), whereas the large proportion of red-staining bacteria in the PDT-treated biofilms confirmed the efficacy of this form of treatment (Fig. 4b, c).

Similar results were obtained in previous studies in which the PDT-induced damage to microbial surfaces was assessed. Sahu et al. (2009) [41] used AFM to visualize the topographical alterations produced by TBO-mediated PDT in *Staphylococcus aureus* and *Escherichia coli*. They observed perturbations to the bacterial wall, bleb formations suggestive of damage to membrane components, and an increase in cell surface roughness. Cheng et al. (2012) [8] evaluated the bactericidal effect of several different laser irradiation methods and PDTs in root canals experimentally infected with *E. faecalis*, using scanning electron microscopy (SEM) to examine the morphology of bacterial cells before and after treatment. SEM revealed the shrunken, rough, and fractured appearance of the bacterial cells that remained after PDT. Melo et al. (2013) [47] used AFM to examine the PDT-induced changes in the shape and size of *Streptococcus mutans* cells. The combination of TBO and LED resulted in a decrease in the diameter of the bacterial cells. Garcez et al. (2013) [48] also demonstrated the effect of PDT to disrupt *Pseudomonas aeruginosa* and *E. faecalis* biofilms in prepared root canals. SEM analysis showed a significant reduction of biofilm after treatment with MB and a diode laser.

The severe perturbations of *E. faecalis* biofilms produced by PDT recommend its use in the eradication of bacterial biofilms. Nevertheless, further studies that include supplemental measuring techniques are needed to explore the effect of PDT on the integrity of microbial biofilms. In addition, whether PDT is equally effective when used on biofilms formed by other microbial species or complex microbial communities remains to be determined. Rates of bacterial dead reported in the literature are highly diverse as experimental conditions are too. Our data are higher than those reported by Soukos and Goodson [2] when they describe that photodynamic therapy killed approximately 63 % of bacteria present in suspension, whereas in biofilms, photodynamic therapy had much less effect reaching 32 % maximal killing. It should be noted that in this work, we have used higher energy fluence and power density. Moreover, it has been shown that PDT efficacy is strain dependent [24].
